# Transdifferentiation of Adipose-Derived Stem Cells into Keratinocyte-Like Cells: Engineering a Stratified Epidermis

**DOI:** 10.1371/journal.pone.0080587

**Published:** 2013-12-02

**Authors:** Claudia Chavez-Munoz, Khang T. Nguyen, Wei Xu, Seok-Jong Hong, Thomas A. Mustoe, Robert D. Galiano

**Affiliations:** Department of Plastic Surgery, Northwestern University, Chicago Illinois, United States of America; Indian Institute of Toxicology Reserach, India

## Abstract

Skin regeneration is an important area of research in the field of tissue-engineering, especially for cases involving loss of massive areas of skin, where current treatments are not capable of inducing permanent satisfying replacements. Human adipose-derived stem cells (ASC) have been shown to differentiate *in-vitro* into both mesenchymal lineages and non-mesenchymal lineages, confirming their transdifferentiation ability. This versatile differentiation potential, coupled with their ease of harvest, places ASC at the advancing front of stem cell-based therapies. In this study, we hypothesized that ASC also have the capacity to transdifferentiate into keratinocyte-like cells and furthermore are able to engineer a stratified epidermis. ASC were successfully isolated from lipoaspirates and cell sorted (FACS). After sorting, ASC were either co-cultured with human keratinocytes or with keratinocyte conditioned media. After a 14-day incubation period, ASC developed a polygonal cobblestone shape characteristic of human keratinocytes. Western blot and q-PCR analysis showed the presence of specific keratinocyte markers including cytokeratin-5, involucrin, filaggrin and stratifin in these keratinocyte-like cells (KLC); these markers were absent in ASC. To further evaluate if KLC were capable of stratification akin to human keratinocytes, ASC were seeded on top of human decellularized dermis and cultured in the presence or absence of EGF and high Ca^2+^ concentrations. Histological analysis demonstrated a stratified structure similar to that observed in normal skin when cultured in the presence of EGF and high Ca^2+^. Furthermore, immunohistochemical analysis revealed the presence of keratinocyte markers such as involucrin, cytokeratin-5 and cytokeratin-10. In conclusion this study demonstrates for the first time that ASC have the capacity to transdifferentiate into KLC and engineer a stratified epidermis. This study suggests that adipose tissue is potentially a readily available and accessible source of keratinocytes, particularly for severe wounds encompassing large surface areas of the body and requiring prompt epithelialization.

## Introduction

The ideal aim of skin regeneration is to find a means to replace or regenerate this complex organ with a normal appearance and with complete functionality [1]–[3]. This process can be done *in-vivo* or *in-vitro* and may require cells, natural or synthetic cell-supporting scaffold materials, bioactive molecules, genetic manipulation, or combination of all of these [4]. Despite the many advances made in epidermal biology, regenerative medicine and tissue engineering, the ideal goal of restoring a functional, cosmetically pleasing skin substitute has remained elusive. Treatments for large acute wounds have not significantly changed in 30 years, and treatments for chronic wounds have only arisen in the past 10 to 15 years. The current “gold-standard” treatment is the split-thickness autograft [5], however burns and severe skin injuries can result in massive skin loss with a lack of available donor sites to perform autografts [6], [7]. The use of cultured allograft skin is limited by the time needed to expand cells from a small biopsy specimen leading to a risk of infection in the burned areas, let alone that it is extremely expensive [8]. Cell-based therapies, which are a branch of regenerative medicine, are a promising area of research that may benefit patients with a need for skin replacement as a result of burn, disease, or trauma [9]. Autologous differentiated cells are commonly studied, however a new wave of research has involved the use of adult stem cells. Adult stem cells have unique features that might represent an effective way to meet the challenges of skin restoration. These include such characteristics as their potential to provide an unlimited source of donor material for grafting, along with their ability to switch into a variety of cell phenotypes *in vitro*.

Human adipose-derived stem cells (ASC) isolated from liposuction or lipectomy specimens have proven their capacity to differentiate into other lineages and cell types *in vitro*. These cells are a subpopulation of the non-adipocyte cell fraction in lipoaspirates, also called the stromal vascular fraction (SVF). Unlike adipocytes, the SVF-cells sediment in aqueous medium and a subset of these cells attach and grow on tissue culture plastic [10]. These cells, as previously shown, have surface antigens similar to those of bone marrow mesenchymal stem cells (BM-MSC) and constitute the ASC subpopulation proper [11].

ASC possess many of the traits common to BM-MSC, including plasticity and a high proliferative potential. ASC can be procured easily from the donor, which makes the goal of clinical application more feasible. Moreover, these cells are derived from adults and therefore circumvent the ethical ambiguities of using embryonic stem cells. Once obtained and differentiated *in-vitro*, these cells can be reintroduced autologously, which has the advantage of eliminating the potential of immune rejection.

However, it has been shown that by three independent research groups that passaged human ASC were not able to stimulate a mixed lymphocyte reaction when co-cultured with allogeneic peripheral blood monocytes [12]–[14]. Similar to BM-MSC [12], the ASC have shown to suppress immune reactions [13], [14]. This indicates that the ASC may not elicit a cytotoxic T-cell response *in-vivo*. Although this observation requires independent and comprehensive testing, if true it would suggest that ASCs may also be useful for allogeneic applications, and may be more readily available to meet clinical needs.

Isolation of ASC yields more stem cells per gram of tissue than from bone marrow [15]. Stem et al., were able to demonstrate that while the number of mesenchymal stem cells (MSC) in bone marrow is approximately 1 in 25,000 to 1 in 100,000 of nucleated cells, the average frequency of ASC in processed lipoaspirate is approximately 2% of nucleated cells. Thus, the yield of ASC from one gram of fat is approximately 5,000 cells, whereas the yield of BM-MSC is 100 to 1,000 cells/mL [16].

As previously mentioned, ASC are a cell population with properties that are very similar, though not identical, to those of BM- MSC [17]–[20]. However, many controversies have risen within different research groups trying to identify a protein panel definitive to characterize these cells, since they lack of a unique cellular marker. The majority of researchers in this field agreed on the following protein expression panel: CD29 [21]–[23], CD49 [24], CD73 [24], CD90 [21]–[24] and CD105 [23]–[28]. There is also consensus that ASC should be negative for endothelial marker CD31 and the hematopoietic marker CD45 [17], [26]. Some discrepancies exist for the expression other proteins. For example, it was very well described that MSC did not express CD34 since this was considered a hematopoietic marker [29], however in the past years, researchers have noticed that freshly isolated MSC do express CD34, however once they are cultured in plastic, CD34 expression is lost [30]. Baer et al recently demonstrated a two subpopulation of ASC that do express the three main markers (CD73, CD90 and CD105) but one was also CD34+ and CD36−; and the second subpopulation was CD34− and CD36+ [31]. For instance, Gronthos and collaborators were able to detect CD34 and CD106 on ASC [32], however Zuk and collaborators did not [33]. Likewise, while Zuk and collaborators were able to detect Stro-1 [33], Gronthos and collaborators did not [32]. Katz and collaborators were able to detect CD117 (c-kit) in ASC from three patients [34], but Tucker and collaborators did not [24]. These discrepancies could reflect differences in cell isolation, methods, how long cells were cultured prior to analysis, the use of monoclonal antibodies detecting different epitopes on the same surface protein, and sensitivity differences between immunohistochemical and flow cytometric detection methods [11], [32], [35], [36].

ASC have also shown extensive proliferative capacity and have shown the ability to undergo differentiation along both mesenchymal lineages (adipogenesis, chondrogenesis, osteogenesis) [22], [37]–[44] and non-mesenchymal lineages (endothelial, smooth muscle, cardiomyocytes, hepatocytes, renal tubular epithelial cells and neuron-like cells), confirming the transdifferentiation ability of ASC [41], [45]–[49]. This versatile differentiation potential places ASC at the advancing front of stem cell-based therapies.

In this study, we hypothesize that ASC might transdifferentiate into keratinocytes (which in this report we describe as keratinocyte-like cells (KLC)) and identify EGF as a critical cytokine responsible for KLC differentiation; we furthermore demonstrate that the KLC can form a stratified epithelium under physiological conditions when seeded on a decellularized dermal matrix.

## Materials and Methods

### Ethics Statement

All human tissue samples were obtained following written informed consent; protocol approved by the institutional review board at Northwestern University. All data was analyzed anonymously and according to the principles expressed in the Declaration of Helsinki.

### Isolation of ASC and Cell Culture

Following informed consent, adipose tissue was obtained from young patients (age: 25±5 years) undergoing elective liposuction. ASC were isolated in accordance with the methods described in previous studies [50]. Briefly, lipoaspirates were washed three times with Hanks balanced salt solution without phenol red (HBSS) (Gibco, Grand Island, NY) and 2% antibiotics (Pen/Step 10,000 U) (Gibco, Grand Island, NY) and incubated in Dispase II (Gibco, Grand Island, NY) (4 mg/mL)/Collagenase I (Gibco, Grand Island, NY) (3 mg/mL) for 2 hrs at 37°C. Following incubation, fetal bovine serum (FBS) (HyClone, Logan UT) was added to a final concentration of 10% to stop the enzymes’ activity and centrifuged at 400×g for 10 min. The pellet was resuspended in HBSS with 2% FBS and 1% antibiotics, followed by serial filtrations using cell strainers (BD Falcon, Bedford, MA) (100 µm, 70 µm and finally 40 µm). Resulting single cells (called Stromal Vascular Fraction, SVF) were then prepared for cell sorting (see below), collected and cultured in a 25 cm^2^ tissue culture flask (10^4^ cells) in DMEM (Cellgro, Manassas, VA) 10%FBS, 1% antibiotics at 37°C in 5% CO_2_. To these final collected cells we denominated ASC. Only sorted ASC were used for this study. The medium was changed every other day until confluent. ASC were detached using 0.25% trypsin- 0.04% EDTA (Gibco, Grand Island, NY) and used for experiments at passages 2–4.

Human foreskin keratinocytes and fibroblasts were obtained from the Skin Disease Research Centre (SDRC) at Northwestern University. Keratinocytes were cultured in Keratinocyte serum-free medium (KSFM) (Gibco, Gran Island, NY) with supplements (Epidermal growth factor plus bovine pituitary extract) (Gibco, Gran Island, NY) 1% antibiotics (Pen/Step 10,000 U) (Gibco, Grand Island, NY) and seeded (1×10^4^ cells) in 25 cm^2^ tissue culture flasks at 37°C in 5% CO_2_. The medium was changed every day until confluent. Keratinocytes were detached using 0.25% trypsin- 0.04% EDTA and used for experiments at passages 2–4. Fibroblalsts were cultured in DMEM (Cellgro, Manassas, VA), 10% FBS (HyClone, Logan UT), and 1% antibiotics (Pen/Step 10,000 U) (Gibco, Grand Island, NY) and seeded (1×10^4^ cells) in 25 cm^2^ tissue culture flasks at 37°C in 5% CO_2_. The medium was changed every other day until confluent. Fibroblasts were detached using 0.25% trypsin- 0.04% EDTA and used for experiments at passages 2–5.

### Flow Cytometry Analysis and Fluorescence-Activated Cell Sorting (FACS)

The SVF was isolated as described above and prepared for flow cytometry analysis and cell sorting as previously described [50]. Briefly, the SVF was incubated with primary antibodies for 45 min at 4°C in supplemented PBS containing 2 mM EDTA and 2% FBS at 1×10^7^ cells per mL. The following primary antibodies were used: anti-human CD90-PE (eBioscience, San Diego, CA), anti-human CD105-APC (eBioscience), anti- human CD31-FITC (eBioscience), anti- human CD45-Krome Orange (eBioscience) and anti- human CD34-ECD (eBioscience). All antibodies were used at 1∶200 dilution according to manufacturer instructions. To assess viability and nuclear staining, cells were stained with propidium iodide (PI) and 4,6-diamidino-2-phenylindole (DAPI) (1 µg mL^−1^). To purify ASC from the SVF, cells had to be double positive for CD90 and CD105 and double negative for CD31 and CD45. Due to some inconsistencies in the literature we analyzed SVF for CD34. Cell analysis and sorting was performed on a MoFlo (Beckman Coulter) equipped with a high-speed multilaser droplet cell sorter fitted with five lasers and a 100-µm nozzle tip at 30 psi. Data analysis was performed using Flowjo software (Treestar, Inc. Ashland, OR). Pure ASC population was sorted from SVF, cultured and expanded as mentioned above. Only sorted ASC were used for all experiments mentioned in this study.

### Adipogenic Differentiation

ASC were further tested in terms of their ability to differentiate into adipogenic lineage [33]. In order to achieve that, ASC were seeded in 12 well plates at a concentration of 4×10^4^ cells/mL and cultured in adipogenic differentiation medium (StemPro, Gibco, Grand Island, NY). After 7 days in culture, cells were fixed in 4% paraformaldehide and intracellular lipid contents were visualized using Oil Red O. Briefly, cells were fixed with 10% formalin for 1 h at room temperature. Then, cells were washed with DI water and prepared by adding 60% isopropanol for 5 min at room temperature. After 5 min, isopropanol was discarded and cells were incubated with Oil Red O working solution (Fisher Scientific), for 5 min at room temperature. Cells were then washed with tap water. Hematoxilin was used as counterstaining.

### Transdifferentiation of Human ASC into Keratinocyte-like Cells (KLC)

To transdifferentiate ASC into keratinocyte-like cells (KLC), we first used the co-cultured system as previously described [51]. ASC (10^5^ cells) were seeded on 6 well plates (bottom chamber) and co-cultured with human keratinocytes (25×10^5^) (upper chamber) using a Trans well insert 3.0 µm pore size (Corning, Corning NY) for 10 days. A mixture of 49% of DMEM, 49% of KSFM with supplements and 2% FBS (test medium) was used to feed the cells in the co-culture systems [52]. Test media was used to feed ASC as negative control. Media was changed every day. Six independent samples were used for transdifferentiation and characterization.

An alternative experiment was performed to transdifferentiate ASC into KLC using the keratinocyte-conditioned media (KCM). Keratinocyes were grown in 75 cm^2^ tissue culture flasks (BD Falcon, Bedford, MA) until confluent. Once reaching confluence, keratinocytes were differentiated using 49.5% KSFM, 49.5% DMEM and 1% antibiotics for up-to 10 days [53]. KCM was collected every day, and centrifuged at 3,000 rpm for 10 min to remove cell debris, and diluted (50∶50) with fresh DMEM 2% FBS. ASC were treated every day with KCM/fresh DMEM (above described) for 10 days. Six independent samples were used for transdifferentiation and characterization.

ASC and KLC were harvested for either RNA extraction or protein extraction, and analyzed using q-PCR or western blot analysis, respectively.

### MTT Assay

To assess cell viability, supernatants from cells in culture (6-well plates) were removed and 500 µl of thyazolyl blue tetrazolium bromide (MTT) was added to each well and incubated for 5 h at 37°C under dark conditions. After 5 h supernatant was removed and 1 mL of Dimethyl sulphoxide (DMSO) (Sigma, Saint Louis, MO) was added to each well and incubated for 5 min at 37°C. Absorbance was read at 570 nm wavelength using a spectrophotometer.

### Western Blot Analysis

After 10 days of ASC being either co-cultured with keratinocytes or treated with KCM, cells were lysed and prepared as previously described [53]. Ten µg were loaded into SDS-PAGE. An anti-involucrin mAb (1∶1000) (Abcam, Cambridge, MA), anti-cytokeratin 5 polyAb (1∶1000) (Abcam), anti-filaggrin mAb (1∶1000) (Leica, Buffalo Grove, IL), or anti-Stratifin polyAb (1∶1000) (Enzo Biomol, Framingdale, NY), were used as primary antibodies. An anti-β actin mAb (1∶30,000) (Sigma, Saint Louis, MO) was used as loading control. The membranes were then incubated with the appropriate horseradish peroxidase-conjugated secondary antibody (Vector, Burlingame, CA) (1∶2500). Immunoreactive proteins were then visualized using ECL Western blotting detection reagent (GE, Buckinghamshire, UK).

### RT-PCR

After 10 days of ASC being either co-cultured with keratinocytes or treated with KCM, total RNA was extracted from cell samples using RNeasy mini kit (Qiagen, Valencia, CA). RNA samples were reverse transcribed using the Superscript**®** first-strand synthesis system (Invitrogen, Carlsbad, CA). For amplification process, the following sense and anti-sense primers shown in Table 1 were used on an AB Prism 7000 PCR System (Applied Biosystems, Foster City, CA). Target gene expression was normalized to β-actin levels, and the comparative cycle threshold (C_t_) method (using the formula: 2^ΔΔCT^) was used to calculate relative quantification of target mRNAs. Keratinocytes were used as positive control and normalized to 1, since they expresses the target mRNA, and Fibroblasts were used as negative control. KLC were first evaluated for the presence of specific keratinoycte genes and compared to untreated ASC. Each assay was performed in triplicate with an *n* of 3–5 independent experiments.

**Table 1:** List of sense and anti-sense primers used for RT-PCR.

Species	Name	Sense	Antisense
Human	KRT5 (Keratin 5)	CAGAGCCACCTTCTGCGTCCTG	GCTGAAGCTACGACTGCCCCC
Human	KRT14 (Keratin 14)	CCTCCTCCAGCCGCCAAATCC	TTGGTGCGAAGGACCTGCTCG
Human	Involucrin	TCCTCCAGTCAATACCCATCAG	CAGCAGTCATGTGCTTTTCCT
Human	Filaggrin	TGAAGCCTATGACACCACTGA	TCCCCTACGCTTTCTTGTCCT
Human	Stratifin	ACTTTTCCGTCTTCCACTACGA	ACAGTGTCAGGTTGTCTCGC
Human	b- actin	AGCCTCGCCTTTGCCGATCC	TTGCACATGCCGGAGCCGTT

### Immunocytofluorescence

Using a tissue slide chamber, ASC were cultured and treated with KCM as described above (under Transdifferentiation of human ASC into keratinocyte-like cells). Then, at different time points (0, 7, 14, 28 and 42), cells were fixed in 4% paraformaldehyde for 10 min at room temperature and then washed with PBS. Non-specific bindings were avoided by using blocking solution (phosphatase-buffered saline solution (PBS) containing 10% goat serum and 5% bovine serum albumin; Sigma). For immunofluorescence microscopy, staining was performed using primary rabbit anti-human cytokeratin-14 (Covance Inc., Princeton, NJ); primary rabbit anti-human Stratifin antibody (Enzo Biomol, Framingdale, NY) and primary mouse anti-human involucrin (Abcam, Cambridge, MA) at 1∶1000 dilution and incubated overnight at 4°C. After washing with PBS three times for 5 min each, samples were incubated with goat anti-rabbit Alexa Fluor 488 or goat anti-mouse Alexa Fluor 594 (Invitrogen, Grand Island, NY) (1∶2500 dilution) at room temperature for 1 hr. Followed by three washes with PBS for 5 min each, samples were incubated with 4,6-diamidino-2-phenylindole (DAPI) (Santa Cruz Biotechnology, Sta. Cruz, CA) for 15 min at room temperature. Finally after three washes with PBS for 5 min each, samples were mounted in Vectashield H-1000 mounting medium for fluorescence (Vector Laboratories Inc. Burlingame, CA). Samples were visualized under a Nikon Eclipse Ti-E microscope (Tokio, Japan) and analyzed using NIS-Elements software (Nikon Corp. Tokio, Japan).

### Quantification of Cytokeratin-14, Stratifin and Involucrin Positive Staining

ASC treated with keratinocyte-conditioned media were stained using anti-human cytokeratin-14, anti-human Stratifin and anti-human Involucrin antibodies as described above. Counterstaining for nuclei was performed using DAPI. Fluorescently stained samples were scanned and data was acquired using an automated system called TissueFAXS system (TissueGnostics Medical and Biotech solutiuons, Viena, Austria). Then, scanned data was analyzed and quantified using TissueQUEST software (TissueGnostics Medical and Biotech solutiuons, Viena, Austria) [54]. Three independent samples were analyzed per time point. Average of positive stained cells were divided by the average number of total number of cells and multiplied by 100, to obtain the percentage.

### Stratification of KLC

The ability to stratify served to evaluate functionality of KLC. To test this, a dermal decellularized matrix, routinely prepared in our laboratory, was used as support to KLC. Briefly, under informed consent, human skin was collected from elective abdominoplasties under the auspices of an IRB-approved protocol. Subcutaneous fat was carefully removed using an industrial razor blade. Skin was disinfected and washed using Betadine and 70% ethanol followed by several washes with PBS (Invitrogen, Carlsbad, CA) and penicillin-streptomycin (Invitrogen, Carlsbad, CA). Skin was then incubated in 1 M NaCl solution overnight to remove the epithelial layer, followed by repetitive freeze and thaw cycles using liquid nitrogen. Further, matrices were incubated in PBS 1% antibiotic (pen/strep) at 4°C for up to 4 weeks. Then matrices were kept at −80°C until use. To further confirm complete decellularization, after 4 weeks matrices were fixed in 10% formalin and paraffin embedded for further sectioning.

After complete decellularization, matrices were placed on the upper chamber of Trans well inserts with a 3.0 µm pore size (Corning, Corning, NY) with the epithelial side facing up. ASC and keratinocytes were seeded on the surface of the matrix at a confluence of 1×10^6^ cell/100 µl/cm^2^. The culture media used for all groups was 49.5% KSFM, 49.5% DMEM and 1% antibiotics, however to achieve transdifferentiation to KLC, we either used KCM in a 1∶1 ratio, or human recombinant epidermal growth factor (EGF) (Gibco) (0.005 µg/ml). After 10 days of culture, Trans well inserts were lifted (air-liquid interphase) allowing cells to differentiate (stratify). Following 10 days post-lifting, the matrix was fixed in 10% formalin for histological evaluation.

### Histological and Immunohistochemical Evaluation

The fixed tissues were immediately embedded in paraffin and sectioned (5 µm thickness). The tissue sections were stained with hematoxylin and eosin (H&E) for histological evaluation. For immunohisotchemical analysis, antigen retrival was performed using Antigen retrival reagent (Dako, Glostrup Denmark), steamed for 20 min. Immunohistochemical staining of cytokeratin-5 (Abcam, Cambridge, MA), cytokeratin-10 (Dako, Carpinteria, CA) and involucrin (Abcam, Cambridge, MA) was achieved using these antibodies at a 1∶200 dilution. Biotinylated secondary antibodes (Vector, Burlingame, CA) were used followed by signal detection using the Vectastain Elite ABC system (Vector, Burlingame, CA) and visualized using 3, 3′-diaminobenzidine. Counterstaining of nucleus was achieved using hematoxylin staining. Images were captured using a Nikon Digital Sight DS-U1 camera system (Nikon, Melville, NY) and processed using Nikon NIS-Elements BR 2.30 software (Nikon).

### Statistical Analysis

Data were expressed as mean ± SD and analyzed with one-way ANOVA using GraphPad Instat Software (GraphPad Software Inc., San Diego, CA). A P-value of less than 0.05 was considered statistically significant.

## Results

### Isolation and Characterization of ASC

**Figure 1:**
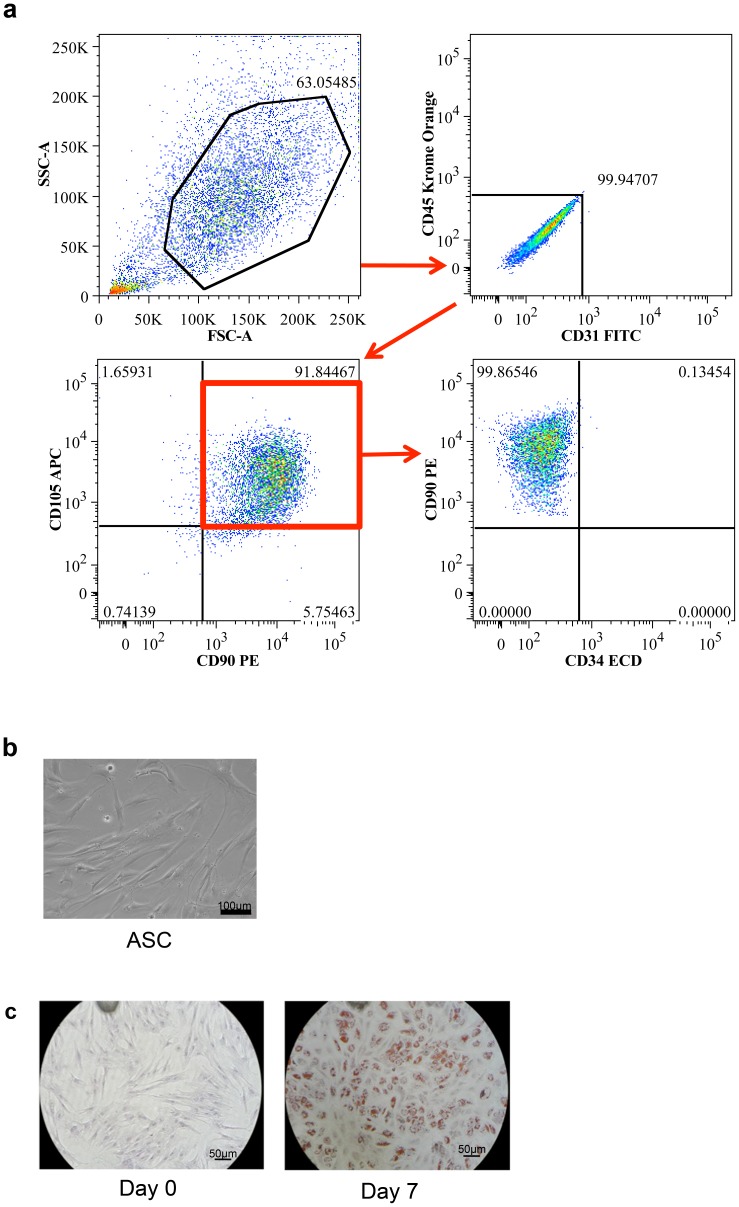
Isolation and characterization of ASC. ASC were isolated and sorted from human fat using fluorescence-activated cell sorting. (a) 92% of the total SVF expressed CD90 and CD105 and were negative for CD31 and CD45. Interestingly these cells (CD90^+^CD105^+^ and CD31^−^CD45^−^) were non-reactive to CD34 antibody. Cells that expressed CD90^+^CD105^+^ and negative for CD31^−^CD45^−^ were sorted, collected and cultured for further experiments. (b) Sorted ASC exhibited a spindle-like shape (80–100 µm in diameter and ∼200 µm in length) Scale bars indicate 100 µm. (c) ASC were cultured under adipogenic conditions for up to 7 days revealing formation of intracellular microdroplets demonstrated by Oil Red O staining. Scale bar indicating 50 µm. All images in this figure were captured with a Nikon digital sight DS-2MBW camera on a Nikon Eclipse TS100 light-microscope at 20x magnification. (Image representative n = 3 independent experiments).

Approximately 20 g of human fat tissue were processed and the SVF was obtained according to our described protocol. SVF cells were prepared for flow cytometry and sorted to obtain a pure ASC population. To delineate the ASC subpopulation from the total SVF, cells that expressed CD90 and CD105 and were negative for CD31 (endothelial) and CD45 (hematopoietic) markers were sorted as ASC [50], [55]. As shown in Fig. 1a, cells were gated according to cell characteristics and viability. From this gated population, less than 0.1% was double negative for CD31 and CD45, and 92% were double positive for CD90 and CD105 (as highlighted with the red frame). These cells were collected for cell culture and further experiments. Interestingly, contrary of what described by some authors [30] and confirming what described by others [29], [56], freshly isolated ASC did not express CD34 (fig. 1a bottom right panel). The sorted ASC were then cultured in DMEM 10% FBS with antibiotics. Throughout the culture period, ASC cells exhibited a spindle-like shape with a wide cytoplasm, and had an approximate size of 80–100 µm in diameter and ∼200 µm in length (Fig. 1b). These results correlate with previous descriptions of these cells [11], [57]. Under adipogenic induction conditions for 7 days, the isolated cells formed intracellular microdroplets demonstrated by staining with Oil Red O. As shown in Fig. 1c, red staining is present in day 7 of culture, but absent in day 0. These results demonstrate that we successfully isolated ASC from liposuction or lipectomy tissues.

### ASC Transdifferentiate into KLC

**Figure 2:**
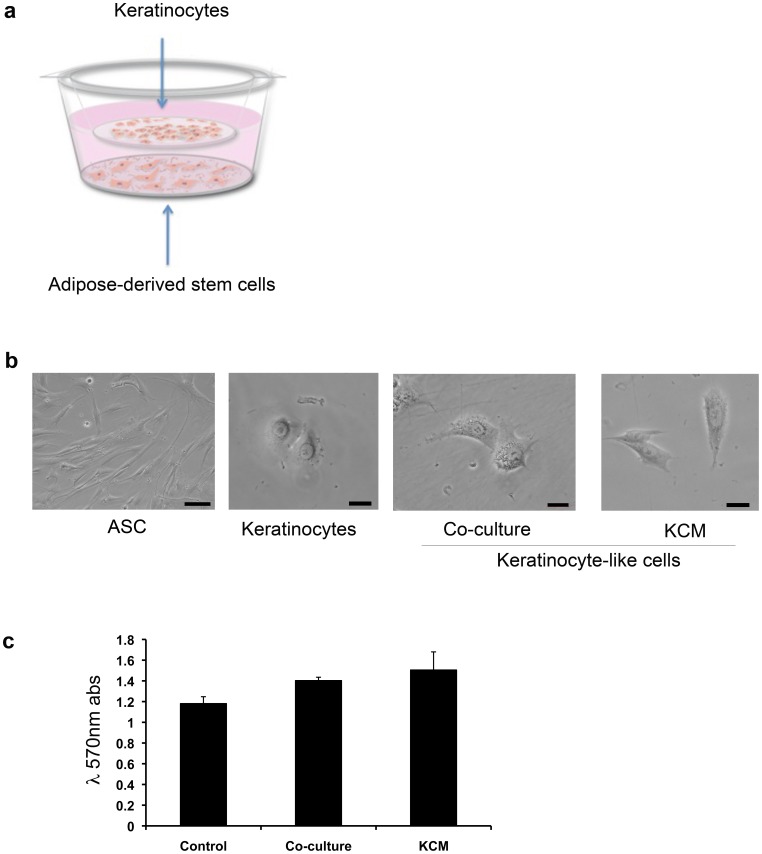
ASC transdifferentiate into KLC. (a) To evaluate ASC capacity to transdifferentiate into KLC ASC were either co-cultured with human keratinocytes or treated with human keratinocyte conditioned media (KCM). (b) Evaluation of the morphology between ASC, keratinocytes and ASC after co-culture or KCM treatment. KLC show a polygonal “cobblestone” shape characteristic of keratinocytes with a size of ∼50 µm in diameter. All pictures captured with a Nikon digital sight DS-2MBW camera on a Nikon Eclipse TS100 light-microscope. ASC panel was captured at 20x, the rest of the panels at 40x magnification. (Scale bars for ASC panel indicate a size of 100 µm, for the rest of the panels; scale bars indicate a size of 20 µm). (c) MTT assay was used to asses cell viability under the two different conditions (co-culture with keratinocytes and KCM treatment). These results demonstrate that either condition in which we induce morphological changes in ASC do not produce any adverse effects on cell viability.

To determine if ASC had the capacity to transdifferentiate into keratinocyte-like cells, two strategies were evaluated. Both are based on the hypothesis that conditioned media contain secreted growth factors and signaling molecules that will have the ability to activate ASC from their quiescent into a differentiated (or transdifferentiated) state. In the first strategy we used a co-culture system growing ASC and keratinocyte cells in a transwell, separated by a membrane, in order to evaluate whether a dynamic cross-communication between these 2 cell types would alter the microenvironment and induce ASC transdifferentiation (Fig. 2a). In the second strategy we added KCM to ASC cells in a culture flask to see if media from keratinocytes by itself would enable ASC transdifferentiation without the need for active crosstalk between the 2 cell types. We first focused our attention on cell morphology. In this regard, ASC in either co-culture with keratinocytes or treated with KCM gradually changed their morphology. After 10 days in culture approximately 20–30% ASC changed their morphology from a “spindle-like” shape with 100 µm diameter and approximately 200 µm in length into a polygonal “cobblestone” shape with a diameter of approximately 50 µm (Fig. 2b). Keratinocytes, as observed in the same figure, also have a polygonal “cobblestone” shape and are approximately 40 µm in diameter, very similar to the transdifferentiated ASC cells. Viability of these cells was assessed using an MTT assay. As shown in Fig. 2c, ASC were viable in either the co-culture system or when treated with KCM. These results suggest that either co-culture with keratinocytes or KCM treatment induces morphological changes in ASC without adverse effects on cell viability.

### KLC Express Keratinocyte Markers

**Figure 3:**
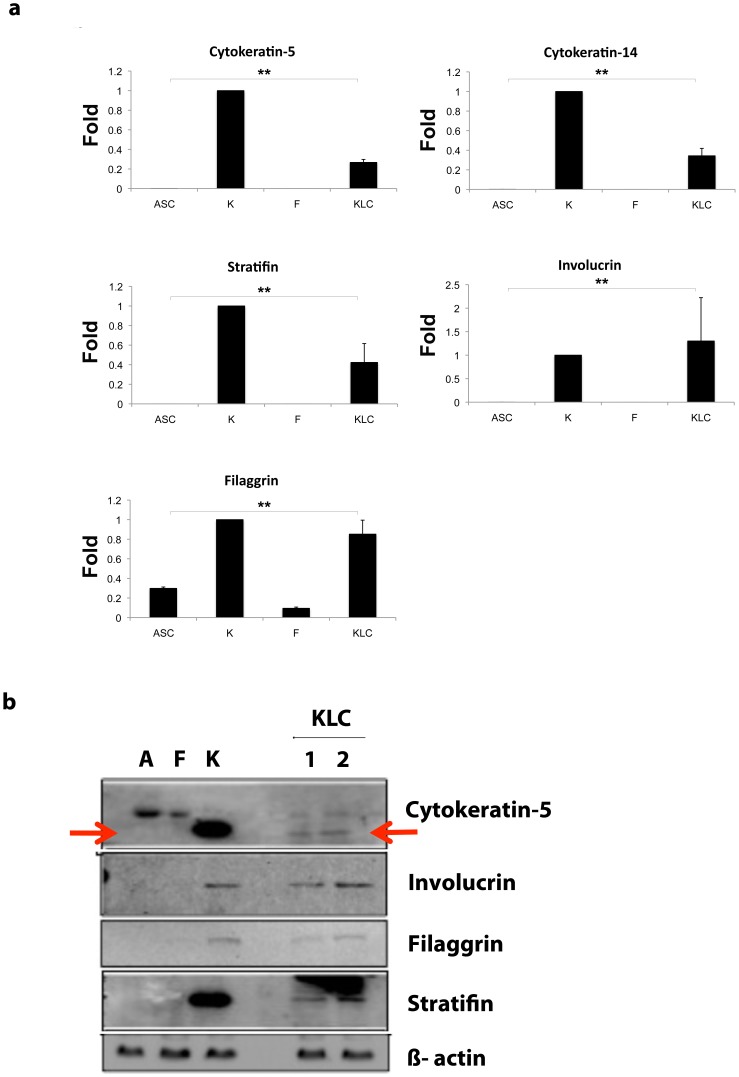
KLC express keratinocyte markers. We evaluated ASC transdifferentiation into KLC by the expression of several keratinocyte markers such as, keratins, involucrin, stratifin and filaggrin. (a) mRNA expression of cytokeratin-5, cytokeratin-14, stratifin, involucrin and filaggrin in ASC, keratinocytes (K), fibroblasts (F), and KLC. Keratinocyte and fibroblasts were used as positive and negative control, respectively. The panel shows the absence with the exception of filaggrin of all other keratinocytes markers in ASC and fibroblasts, and the positive expression of these markers in KLC after ASC-transdifferentation, (Cytokeratin-5, 0.26±0.03 vs. 0.0008±0.0001; cytokeratin-14, 0.34±0.07 vs 0.001±0.000089; stratifin, 0.42±0.19 vs 0.000055±0.0000056 and involucrin, 1.3±0.9 vs 0.0008±0.0005; respectively). In the case of filaggrin, KLC showed almost a 3 fold up-regulation when compared to that of ASC (0.85±0.14 vs 0.29±0.01; respectively) **p<0.01; n = 3 independent experiments. Keratinocytes were normalized to 1 since these cells possess these markers. Statistical analysis was obtained comparing KLC to ASC. (b) Western blot analysis confirmed the mRNA findings. KLC showed the presence of cytokeratin-5, involucrin, filaggrin and stratifin proteins comparable to those from keratinocyte lysates, however no bands were detected in ASC or fibroblast lysates. (Arrows indicate Cytokeratin-5 band, according to its molecular weight. Upper bands may correspond to unspecific binding at a higher molecular weight not corresponding with cytokeratin-5 molecular weight) (Image representative n = 3 independent experiments).

To demonstrate the progressive epithelial determination of ASC, multiple specific markers for keratinocytes (keratins, involucrin, stratifin and filaggrin) were selected and evaluated using q-PCR and Western blot analysis. Neonatal human keratinocytes and dermal fibroblasts were used as positive and negative controls respectively. Keratinocytes samples were normalized to one as an mRNA expression reference. As shown in Fig. 3a, with exception of Filaggrin, ASC did not express any of the keratinocyte differentiation markers. Interestingly, KLC showed the expression of cytokeratin-5, cytokeratin-14, stratifin and involucrin at the mRNA level when compared to that of ASC (Cytokeratin-5, 0.26±0.03 vs. 0.0008±0.0001; cytokeratin-14, 0.34±0.07 vs 0.001±0.000089; stratifin, 0.42±0.19 vs 0.000055±0.0000056 and involucrin, 1.3±0.9 vs 0.0008±0.0005; respectively) (**p<0.001; n = 3 independent experiments). Likewise, KLC showed almost a 3-fold up-regulation of filaggrin when compared to that of ASC (0.85±0.14 vs 0.29±0.01; respectively; **p<0.001; n = 3 independent experiments). Overall, KLC demonstrated gene expression profiles of the keratinocyte-defining markers similar to those of keratinocytes. In addition, to confirm our mRNA results, protein was extracted from control ASC, keratinocytes, fibroblasts and KLC and analyzed using Western blot for similar keratinocyte-specific markers. As shown in Fig. 3b, cytokeratin-5, involucrin, and stratifin were expressed in KLC and keratinocyte lysates but not in ASC or fibroblasts.

**Figure 4:**
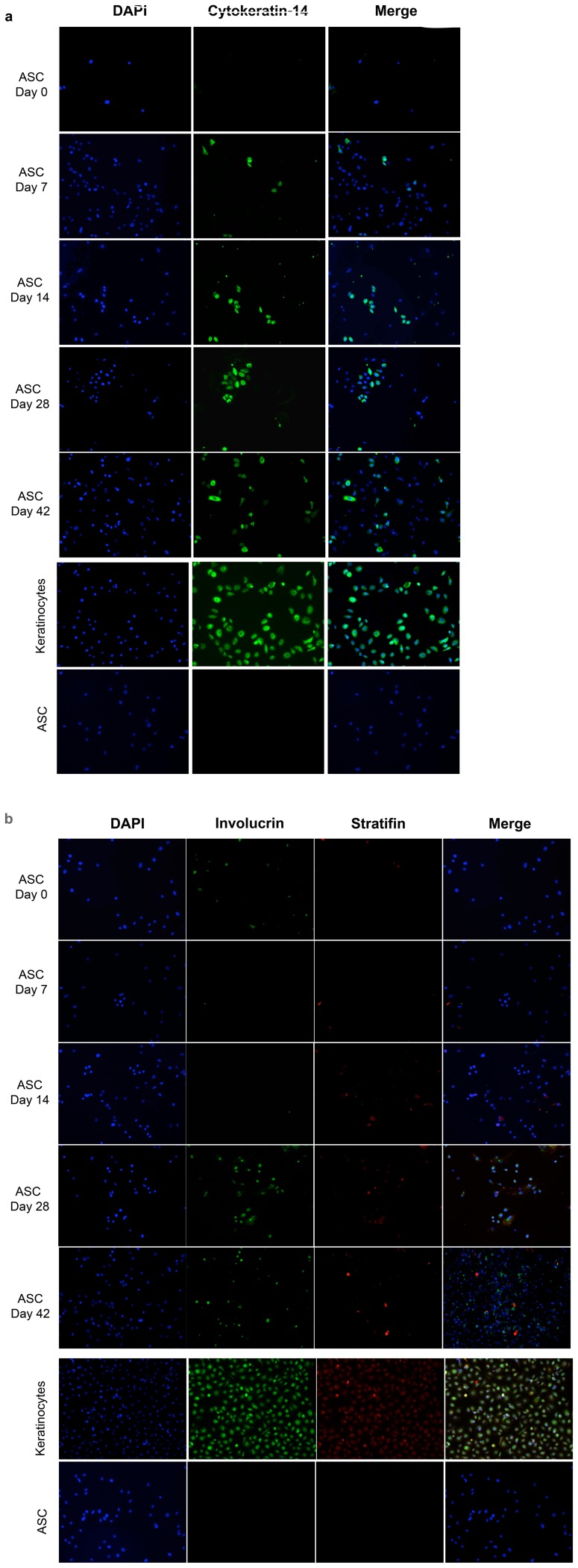
Transdifferentiation of ASC into KLC at different time points. ASC were seeded in chamber slides and cultured in the presence of KCM. Cells were fixed and stained at different time points (Days 0, 7, 14, 28 and 42) for evaluation. (a) ASC were immunostained with anti-human cytokeratin-14. DAPI was used as counterstaining. Images demonstrate detection of cytokeratin-14 as early as day 7 post-treatment. Keratinocytes and ASC were used as positive and negative controls, respectively. (Image representative n = 3 independent experiments) (b) ASC were immunostained with anti-human Involucrin and anti-human Stratifin. DAPI was used as counterstaining. Expression of stratifin was detected starting at day 14. Expression of involucrin was observed starting at day 28. Keratinocytes and ASC were used as positive and negative controls, respectively. (Image representative n = 3 independent experiments). (Table 2) Quantification of transdifferentiated ASC into KLC. ASC cells were seeded on chamber slides and cultured in the presence of KCM. At different time points (Days 0, 7, 14, 28, and 42) cells were fixed and stained using anti-human cytokeratin-14, anti-human Stratifin and anti-human Involucrin. Slides were scanned using TissueFAXS system and quantified using TissueQuest software. Average of positive stained cells were divided by the average of total number of cells (nuclei counts) and multiplied by 100, to obtain the percentage. (*n = 3 slides per time point).

Furthermore, we wanted to evaluate and quantify the percentage of ASC transdifferentiating into KLC. In order to achieve this, we used cytokeratin-5/14 as a transdifferentiating marker since it is a marker for basal keratinocytes. ASC were seeded on chamber slides and treated with KCM through out different time points (0, 7, 14, 28 and 42 days). Keratinocytes and ASC were used as positive and negative controls, respectively. Cells were immunostained for Cytokeratin-5/14, Stratifin and Involucrin. As shown in fig. 4a and b, as early as 7 days we start seeing positive staining for cytokeratin-14. However, 14 days are required to see positive staining for Stratifin and 28 days for Involucrin. Quantification was carried out using an automated system (TissueGnostics) that consists in scanning the entire slide (TissueFax) and using a software (TissueQuest), which automatically quantifies the amount of positive cells with respect to the number of total cells (nuclei staining: DAPI). As shown in Table 2, at day 0 we did not observe any positive cytokeratin-14 staining, at day 7 approximately 5% (5±0.38; n = 3) of cells showed positive cytokeratin-14 stain, at day 14 approximately 15% (15±1.8; n = 3) showed positive stain, by day 28 approximately 23% (23±2.6; n = 3) showed positive stain, and at day 42 approximately 30% (30±2.8; n = 3) showed positive cytokeratin-14 stain. Cells started showing positive staining for Statifin at day 14, approximately 3% (3±0.8; n = 3); at day 28 approximately 8% (8±0.6; n = 3) of cells showed positive staining and by day 42 approximately 10% (10±0.9; n = 3) were positively stained. Involucrin showed a different pattern, we could not observe positive staining at day 14, however by day 28 approximately 18% (18±2.0; n = 3) of the cells were positively stained, and by day 42 approximately 21% (21±1.2; n = 3) were positively stained. These results confirm the fact that Cytokeratin-14 is a basal keratinocyte marker, which suggests that ASC first transdifferentiate to a basal keratinocyte-like cell and as they mature they become a differentiated keratinocyte-like cell, showed by the presence of Stratifin and Involucrin markers. These results led us to think that KLC were able to stratify since these results were showing differentiation.

**Table 2:** 

Time Points (Days)	Transdifferentiation Rate by Quantificationof Cytokeratin-14 (Approx. %)*	Quantification of Stratifin(Approx. %)*	Quantification of Involucrin(Approx. %)*
0	0	0	0
7	5±0.38	0	0
14	15±1.8	3±0.8	0
28	23±2.6	8±0.6	18±2.0
42	30±2.8	10±0.9	21±1.2

* n = 3 sides per time point.

Over all, these results revealed not only phenotypic changes of ASC as shown in fig. 2, but also genotypic changes in ASC, demonstrating their capacity to transdifferentiate into KLC and possibly to form a stratified epidermis-like structure.

### KLC have the Capacity to Stratify on a Dermal Scaffold

**Figure 5:**
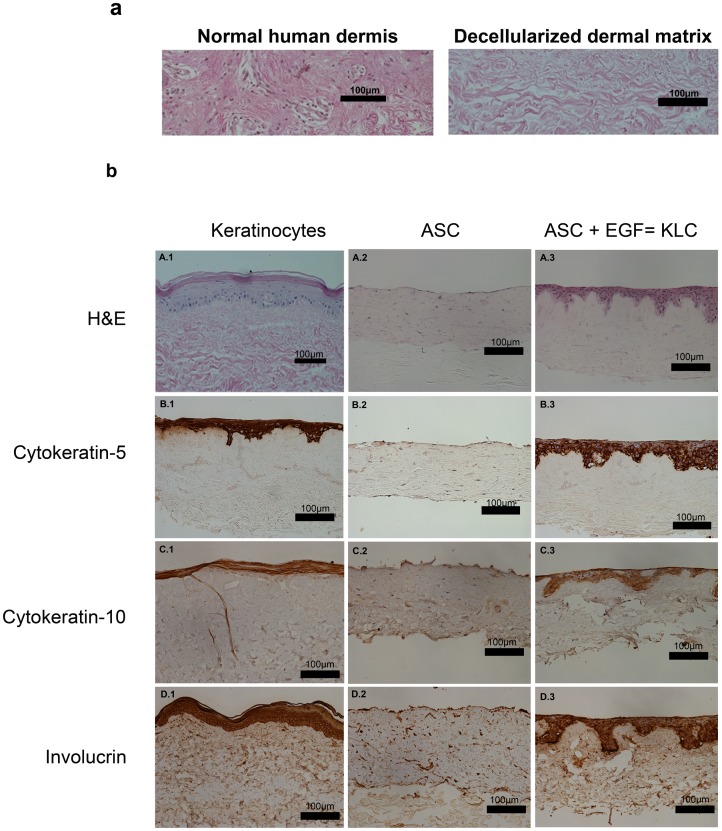
KLC have the capacity to stratify. (a) Human skin was decellularized and confirmed complete cell removal by H&E staining. (b) ASC were seeded on the epithelial side of human dermal decellularized matrices in the presence or absence of EGF (0.005 µg/mL). Human dermal decellularized matrices seeded with keratinocytes were used as controls. All three groups were kept in the same media conditions and lifted to an air-liquid interphase at the same time. Histological analysis revealed the presence of several layers of KLC with an organization similar to that of keratinocytes (A.1and 3). Surprisingly, ASC without EGF not only did not transdifferentiate into KLC, but instead migrated inside the matrix (A.2). Immunohistochemical analysis revealed a positive staining of cytokeratin-5 (B.3), cytokeratin-10 (C.3) and involucrin (D.3) in stratified KLC matrices comparable to that of the stratified keratinocyte matrix (A, B, C and D.1). No positive staining was found in the ASC seeded matrices (A, B, C and D.2). Images captured using Nikon Digital Sight DS-5M on a Nikon Eclipse 50i microscope at 20x magnification. Scale bars indicate 100 µm (Image representative n = 3 independent experiments).

In preliminary experiments, human skin-derived acellular dermal matrices were evaluated for complete decellularization using H&E staining, confirming the absence of cells in the matrix (Fig. 5a). This substrate was then utilized to further evaluate if KLC had the potential to form a three-dimensional stratified construct. This is a critical assay as keratinocyte stratification and differentiation is a hallmark of normal epidermal development. All cells (keratinocytes and ASC) were seeded on the epidermal side of a decellualrized dermal matrix in the presence or absence of EGF (0.005 µg/ml). The media used for all groups was a mixture of DMEM and KSFM (50∶50), containing a higher Ca^2+^ concentration [1.8 mM] (however, still under physiological conditions), which has previously been demonstrated to induce keratinocyte differentiation [53]. Three groups were evaluated: 1) keratinocytes seeded on a decellularized dermal matrix in the presence of EGF (0.005 µg/ml) used as control (labeled keratinocytes), 2) ASC seeded on a decellularized dermal matrix in the absence of EGF (labeled ASC) and, 3) ASC seeded on a decellularized dermal matrix in the presence of EGF (0.005 µg/ml) (labeled ASC+EGF). All groups were lifted to an air-liquid interphase as described in the Materials and Methods section. After the development of a stratified epidermis, evaluated by light microscopy (images not shown), tissues were fixed and embedded in paraffin for hematoxilin and eosin staining (H&E) as well as for immunohistochemistry evaluation. H&E staining (Fig. 5b, panels A1–3) revealed the presence of several layers of KLC with an organization very similar to that formed by keratinocyte cells. In contrast, non-induced ASC not only were unable to stratify, but instead migrated into the matrix. Furthermore, the tissues were evaluated for the presence of other keratinocyte markers such as cytokeratin-5, cytokeratin-10 and involucrin. Cytokeratin-5 (K-5) staining (panels B1–3) revealed the presence of positive staining in stratified KLC tissue comparable to that of the stratified keratinocyte tissue. As expected there was no positive K-5 staining in the non-induced ASC tissue. Very similar results were found for cytokeratin-10 (K-10) staining (panels C1–3). Stratified KLC tissue demonstrated strongly positive staining comparable to that of stratified keratinocyte tissue. Non-induced ASC tissue did not show positive staining for K-10. Involucrin staining (panels D1–3) showed a positive signal in stratified KLC tissue and at a comparable intensity to stratified keratinocytes tissue. However, no signal was present in the non-induced ASC tissue.

These results demonstrate that ASC have the capacity to transdifferentiate and stratify when seeded onto a decellularized dermal matrix and treated with EGF (0.005 µg/ml); revealing a very similar morphology to that of native epidermis.

## Discussion

Adipose-derived stem cells are an easily harvested, readily available, and relatively prevalent (1 g of fat yields 5,000 ASC) [16] form of an adult mesenchymal stem cell. Unfortunately there is no unique marker that can identify and separate ASC from other cell types exactly [58]. Therefore a combination of markers is needed to purify ASC from the SVF. In this study, we determined ASC as cells expressing CD90 and CD105 and negative for CD31 and CD45. We further analyzed ASC for the presence of CD34 protein as reported by some investigators [30]–[32]. Surprisingly, CD34 was not expressed in neither freshly isolated (Fig. 1a) nor cultured ASC. It is possible that as described by Baer and collaborators we have isolated a subpopulation of ASC which is CD34^−^ [31]. One can also speculate that these subpopulations can vary depending on the location of which these cells have been isolated [59]. It is well known that fat tissue contains a heterogeneous population of cells, such as adipocytes, endothelial cells, periocytes, adventitial stromal cell-like cells [60], among others that we may have not identified yet. For example Lin and collaborators proposed that MSC intimately associated with the vasculature, expressing CD34 should be called vasculature stem cells (VSC). They also recognize the possibility of certain MSC not perivascularly associated making certain subsets of MSC indeed CD34− [30]. Despite this controversy, CD34 expression or not does not seem to affect the therapeutic efficacy of MSC.

ASC also share several defining traits common to other adult mesenchymal stem cell types, including plasticity, a high proliferative potential, the ability to secrete protective bio-active molecules and a capacity to modulate immune responses [55], [61]. Due to all these characteristics, we evaluated the potential of ASC as an alternative cell type from which to derive an epidermis [19]. Recent findings have suggested that ASC can not only differentiate into cell types from the mesenchyme germ layer, but, as we have also shown in this study, they are also able to differentiate into cell types from different germ layers, a phenomenon termed “transdifferentaition” [62], [63]. Previous reports have stated that the key factor for initiating differentiation is the change of the cell microenvironment either with a defined media containing lineage-specific differentiation factors or with a co-culture method [64]. Here in this study, we have demonstrated for the first time that human ASC have the capacity to transdifferentiate into keratinocyte-like cells by three methods: 1) using a co-culture system, 2) by treating ASC with keratinocyte conditioned media and 3) by induction with a specific growth factor (EGF) and high Ca2+ concentration (within physiological conditions) (Fig. 2, 3, 4 and 5). Stem cell transdifferentiation *in-vitro* and *in-vivo* have been reported by many research groups, nevertheless it is still debatable whether transdifferentiation occurs *in-vivo* in settings such as the post-injury milieu. Concerns including methodologic questions regarding the intensity and specificity of cell labeling, accuracy of cell isolation, the low number of transdifferentiated stem cells detected, and variable results obtained by different research groups have engendered skepticism about the reproducibility as well as physiologic relevance of these findings [65]. A low number of transdifferentiated cells has been a common denominator for all previous reports (10–20% transdifferentiation) [65]; in this study we observed a 30% rate of ASC transdifferentiation into KLC by day 42. This was higher than expected when compared to other reports (Table 2).

In addition, we have demonstrated that these transdifferentiated KLC express specific keratinocyte markers, such as cytokeratins (5, 10 and 14), stratifin, involucrin and filaggrin (Fig. 3). Cytokeratin-5/14 is an epithelial basal layer marker, reason why we chose this as the transdifferentation marker for our experiments. Cytokeratin-10 is an early marker of epidermal differentiation expressed in all suprabasal cell layers including the stratum corneum. Stratifin, also known as epithelial cell marker, is a keratinocyte 14-3-3 specific protein which is expressed in differentiatied keratinocytes. Filaggrin is an intermediate marker of epidermal differentiation expressed strictly in well-differentiated keratinized epithelial cells. It is known to be an essential protein for the regulation of epidermal homeostasis. Involucrin is the terminal marker of epidermal differentiation and is expressed in the upper spinous and granular layers of human skin [66], [67]. This study showed the presence of all these markers in the transdifferentiated KLC derived from ASC at the protein as well as at the mRNA levels as early as 7 days in culture (Fig. 3). Furthermore, we studied the transdifferentiation rate in ASC into KLC using cytokeratin 5/14 antibody as a transdifferentiation marker though out 42 days (Fig. 4a). Automated quantification of positive cells demonstrated a 5% rate transdifferentiation at day 7 and a maximum rate of 30% by day 42 (Table 2).

To prove the functionality of these ASC-derived cells using an assay that defines epithelial behavior, we seeded them on a human decellularized dermal matrix for ten days in the presence of [1.8 mM] Ca^2+^ and EGF to induce transdifferentiation and then lifted the epidermis to an air-liquid interphase to induce keratinocyte differentiation and stratification. EGF has been previously reported to be able to induce epithelial transdifferentiation in bone marrow-derived mesenchymal stem cells (BM-MSC) [68]. As shown in our study, EGF in combination with an increased Ca2+ concentration was sufficient and necessary to induce transdifferentiation of ASC into keratinocyte-like cells (Fig. 5) in our model system. Further studies need to be done to examine the mechanism through which EGF is able to induce transdifferentiation in ASC. Recent studies have shown that early growth response-1 (EGR-1) is highly expressed in MSC, and that EGF strongly upregulates EGR-1 and also EGF receptor in a protein kinase C-and/or mitogen-activated protein kinase-extracellular-signal-regulated kinase-dependent manner [69]. Confirmation of this finding is required in the ASC. In addition, as previously shown, high levels of Ca^2+^ ([1.8 mM]) in the media were able to induce keratinocyte differentiation [53]. As is well known, Ca^2+^ is one of the essential factors inducing this process, due to the natural Ca^2+^ gradient present in the epidermis [70]. Transdifferentiation as well as KLC differentiation were demonstrated by showing the presence of keratinocyte and keratinocyte-differentiation markers, as shown in Fig. 4 and 5. As mentioned above, proteins like involucrin, cytokeratin-10 and filaggrin are specific keratinocyte differentiation markers, whereas cytokeratin-5/14 is specific to the basal layer. It is also pertinent that the process of ASC isolation, culture transdifferentiation and stratification occurred over a span of 20 days, which suggests that the process identified in this report may have significant clinical relevance once further refined. Currently we are looking into ways of expanding and storing KLC and testing its functionality after long periods of time.

In conclusion, our study demonstrates that ASC not only posses the capacity to transdifferentiate into keratinocyte-like cells (KLC) but also these KLC are able to differentiate and stratify in a physiologically relevant 3-D organotypic culture system. These findings suggest that ready available ASC may potentially be useful for cellular therapies providing expeditious epidermal cover in cases of massive skin loss due to burns or trauma.

## References

[1] Hollander A, Macchiarini P, Gordijn B, Birchall M (2009) The first stem-cell based tissue-engineered organ replacement: implications for regenerative medicine and society. Regen Med 4: 147–148. 19317632 10.2217/17460751.4.2.147

[2] Orlando G, Baptista P, Birchall M (2011) Regenerative medicine as applied to solid organ transplantation: current status and future challenges. Transplant Int 24: 223–232. 10.1111/j.1432-2277.2010.01182.x PMC3817209 21062367

[3] Orlando G, Wood KJ, Stratta R (2011) Regenerative medicine and organ transplantation: past, present and future. Transplantation 91: 1310–1317. 21505379 10.1097/TP.0b013e318219ebb5

[4] Atala A (2009) Engineering organs. Curr Opin Biotechnol 20: 575–592. 19896823 10.1016/j.copbio.2009.10.003

[5] Lineen E, Namias N (2008) Biologic dressing in burns. J Craniofac Surg 19: 923–928. 18650713 10.1097/SCS.0b013e318175b5ab

[6] Atiyeh BS, Gunn SW, Hayek S (2005) State of the art in burn treatment. Wold J Surg 29: 131–148. 10.1007/s00268-004-1082-2 15654666

[7] Priya SG, Jungvid H, Kumar A (2008) Skin tissue engineering for tissue repair and regeneration. Tissue Eng Part B Rev 14: 105–118. 18454637 10.1089/teb.2007.0318

[8] Boyce ST, Kagan RJ, Meyer N (1999) The 1999 clinical research award: cultured skin substitutes combined with Integra Artificial Skin to replace native skin autograft and allograft for the closure of excised full-thickness burns. J Burn Care Rehabil 20: 453–461. 10613682 10.1097/00004630-199920060-00006

[9] Orlando G, Wood KJ, De Coppi P, Baptista P, Binder KW, et al (2012) Regenerative medicine as applied to general surgery. Ann Surg 255: 867–880. 22330032 10.1097/SLA.0b013e318243a4db PMC3327776

[10] Verfaillie C (2005) Stem cell plastcity. Hematology 10: 293–296. 16188690 10.1080/10245330512331390113

[11] Zuk PA, Zhu M, Mizuno H (2001) Multilineage cells from human adipose tissue: implications for cell-based therapies. Tissue Eng 7: 211–228. 11304456 10.1089/107632701300062859

[12] Cui L, Yin S, Yang P, Liu B, Zhang Y, et al (2005) Human adipose derived stem cells suppress lymphocyte proliferation induced by cellular or nonspecific mitogenic stimuli. Zhonghua Yi Xue Za Zhi 85: 1890–1894. 16255982

[13] Puissant B, Barreau C, Bourin P, Clavel C, Corre J, et al (2005) Immunomodulatory effect of human adipose tissue-derived adult stem cells: comparison with bone marrow mesenchymal stem cells. Br J Haematol 129: 118–129. 15801964 10.1111/j.1365-2141.2005.05409.x

[14] McIntosh K, Zvonic S, Garrett S, Mitchell JB, Floyd ZE, et al (2006) The immunogenicity of human adipose derived stem cells: temporal changes in vitro. Stem Cells 24: 1245–1253. 10.1634/stemcells.2005-0235 16410391

[15] Brayfield CA, Marra KG, Rubin J (2010) Adipose tissue regeneration. Curr Stem Cell Res Ther 5: 116–121. 19941458 10.2174/157488810791268582

[16] Strem BM, Hicok KC, Zhu M, Wulur I, Alfonso Z, et al (2005) Multipotential differentiation of adipose tissue-derived stem cells. Keio J Med 54: 132–141. 16237275 10.2302/kjm.54.132

[17] De Ugarte DA, Alfonso Z, Zuk P, Elbarbary A, Zhu M, et al (2003) Differential expression of stem cell mobilization-associated molecules on multi-lineage cells from adipose tissue and bone marrow. Immunol Lett 89: 267–270. 14556988 10.1016/s0165-2478(03)00108-1

[18] Rodriguez AM, Elabd C, Amri EZ, Ailhaud G, Dani C (2005) The human adipose tissue is a source of multipotent stem cells. Biochimie 87: 125–128. 15733747 10.1016/j.biochi.2004.11.007

[19] Rodriguez AM, Pisani D, Dechesne CA, Turc-Carel C, Kurzenne JY, et al (2005) Transplantation of a multipotent cell population from human adipose tissue induces dystrophin expression in the immunocompetent mdx mouse. J Exp Med 201: 1397–1405. 15867092 10.1084/jem.20042224 PMC2213197

[20] Wickham MQ, Erickson GR, Gimble J, Vail TP, Guilak F (2003) Multipotent stromal cells derived from the infrapatellar fat pad of the knee. Clin Orthop Relat Res 412: 196–212. 10.1097/01.blo.0000072467.53786.ca 12838072

[21] Fraser JK, Schreiber R, Strem B, Zhu M, Alfonso Z, et al (2006) Plasticity of human adipose stem cells towards endothelial cells and cardiomyocytes. Nat Clin Pract Cardiovasc Med 3: S33–S37. 16501628 10.1038/ncpcardio0444

[22] Fraser JK, Wulur I, Alfonso Z, Hedrick M (2006) Fat tissue: an underappreciated source of stem cells for biotechnology. Trends Biotechnol 24: 150–154. 16488036 10.1016/j.tibtech.2006.01.010

[23] Lee R, Kim B, Choi H, Suh K, Bae Y, et al (2004) Characterization and expression analysis of mesenchymal stem cells from bone marrow and adipose tissue. Cell Physiol Biochem 14: 311–324. 15319535 10.1159/000080341

[24] Tucker H, Bunnell B (2011) Characterization of human adipose-derived stem cells using flow cytometry. In: Gimble JM, Bunnell BA editor. Adipose-derived stem cells: Methods and Protocols. New York: Springer Science. 121–131. 10.1007/978-1-61737-960-4_10 21082399

[25] Dennis JE, Carbillet JP, Caplan AI, Charbord P (2002) The STRO-1+ marrow cell population is multipotential. Cell Tissue Organs 170: 73–82. 10.1159/000046182 11731697

[26] Gronthos S, Franklin DM, Leddy HA, Robey PG, Storms RW, et al (2001) Surface protein characterization of human adipose tissue -derived stromal cells. J Cell Physiol 189: 54–63. 11573204 10.1002/jcp.1138

[27] Majumdar MK, Thiede MA, Mosca JD, Moorman M, Gerson SL (1998) Phenotypic and functional comparison of cultures of marrow-derived mesenchymal stem cells (MSC) and stromal cells. J Cell Physiol 176: 57–66. 9618145 10.1002/(SICI)1097-4652(199807)176:1<57::AID-JCP7>3.0.CO;2-7

[28] Simmons PJ, Gronthos S, Zannetino A, Ohta S, Graves S (1994) Isolation, characterization and functional activity of human marrow stromal progenitors in hemopoiesis. Prog Clin Biol Res 389: 271–280. 7700911

[29] Dominici M, Le Blanc K, Mueller I, Slaper-Cortenbach I, Marini F, et al (2006) Minimal criteria for defining multipotent mesenchymal stromal cells. Cytotherapy 8: 315–317. 16923606 10.1080/14653240600855905

[30] Lin C, Ning H, Lin G, Lue T (2012) Is CD34 a truly negative marker for mesenchymal stromal cells? Cytotherapy 14: 1159–1163. 23066784 10.3109/14653249.2012.729817 PMC3846603

[31] Baer P, Kuci S, Krause M, Kuci Z, Zielen S, et al (2013) Comprehensive phenotypic characterization of human adipose-derived stromal/stem cells and their subsets by a high throughput technology. Stem Cells Dev 22: 330–339. 22920587 10.1089/scd.2012.0346

[32] Gronthos S, Franklin D, Leddy H (2001) Characterization of surface protein expression on human adipose tissue-derived stromal cells. J Cell Physiol 189: 54–63. 11573204 10.1002/jcp.1138

[33] Zuk P, Zhu M, Ashjian P, De Ugarte D, Huang J, et al (2002) Human adipose tissue is a source of multipotent stem cells. Mol Biol Cell 13: 4279–4295. 12475952 10.1091/mbc.E02-02-0105 PMC138633

[34] Katz A, Tholpady A, Tholpady S, Shang H, Ogle R (2005) Cell surface and transcriptional characterization of human adipose-derived adherent stromal (hADAS) cells. Stem Cells 23: 412–423. 15749936 10.1634/stemcells.2004-0021

[35] William S, Rose D, Jarell B (1994) Liposuction derived human fat used for vascular sodding contains endothelial cells and not mesothelial cells as the major cell type. J Vasc Surg 19: 916–923. 8170048 10.1016/s0741-5214(94)70019-2

[36] Young C, Jarrell B, Hoying J (1992) A porcine model for adipose tissue-derived endothelial cell transplantation. Cell Transplant 1: 293–298. 1285346 10.1177/096368979200100406

[37] Erickson GR, Gimble JM, Franklin DM, Rice HE, Awad H, et al (2002) Chondrogeneic potential of adipose tissue-derived stromal cells in vitro and in vivo. Biochem Biophys Res Commun 290: 763–769. 11785965 10.1006/bbrc.2001.6270

[38] Halvorsen YD, Franklin DM, Bond AL, Hitt DC, Auchter C, et al (2001) Extracellular matrix mineralization and osteoblast gene expression by human adipose tissue-derived stromal cells. Tissue Eng 7: 729–741. 11749730 10.1089/107632701753337681

[39] Jiang Y, Jahagirdar BN, Reinhardt RL, Schwartz RE, Keene CD, et al (2002) Pluriotency of mesenchymal stem cells derived from adult marrow. Nature 418: 41–49. 12077603 10.1038/nature00870

[40] Nagaya N, Kangawa K, Itoh T, Iwase T, Murakami S, et al (2005) Transplantation of mesenchymal stem cells improves cardiac function in a rat model of dilated cardiomyopathy. Circulation 112: 1128–1135. 16103243 10.1161/CIRCULATIONAHA.104.500447

[41] Safford KM, Hicok KC, Safford SD, Halvorsen YD, Wilkison WO, et al (2002) Neurogenic differentiation of murine and human adipose-derived adult stromal cells. Biochem Biophys Res Commun 294: 371–379. 12051722 10.1016/S0006-291X(02)00469-2

[42] Xu Y, Malladi P, Wagner DR, Longaker MT (2005) Adipose-derived mesenchymal cells as a potential cell source for skeletal regeneration. Curr Opin Mol Ther 7: 300–305. 16121695

[43] Yang L, Zheng J, Liu X, Hui G, Fei J, et al (2003) Adipose tissue-derived stromal cells differentiate into neuron-like cells. Sichuan Da Xue Xue Bao Yi Xue Ban 34: 381–384. 12910668

[44] Tsuji W, Inamoto T, Yamashiro H, Ueno T, Kato H, et al (2009) Adipogenesis induced by human adipose tissue-derived stem cells. Tissue Eng Part A 15: 83–93. 18759663 10.1089/ten.tea.2007.0297

[45] Safford KM, Safford SD, Gimble JM, Shetty AK, Rice HE (2004) Characterization of neuronal/glial differentiation of murine adipose-derived adult stromal cells. Exp Neurol 187: 319–328. 15144858 10.1016/j.expneurol.2004.01.027

[46] Qian DX, Zhang HT, Ma X, Jiang XD, Xu R (2010) Comparison of the efficiencies of three neural induction protocols in human adipocyte stromal cells. Neurochem Res 35: 572–579. 19960248 10.1007/s11064-009-0101-y

[47] van Dijk A, Niessen HW, Zandieh Doulabi B, Visser FC, van Milligen FJ (2008) Differentiation of human adipocyte-derived stem cells towards cardiomyocytes is facilitated by laminin. Cell Tissue Res 334: 457–467. 18989703 10.1007/s00441-008-0713-6

[48] Lue J, Lin G, Ning H, Xiong A, Lin CS, et al (2010) Transdifferentiation of adipocyte-derived stem cells into hepatocytes: a new approach. Liver Int 30: 913–922. 20353420 10.1111/j.1478-3231.2010.02231.x

[49] Baer PC, Bereiter-Hahn J, Missler C, Brzoska M, Schubert R, et al (2009) Conditioned medium from renal tubular epithelial cells initiates differentiation of human mesenchymal stem cells. Cell Prolif 42: 29–37. 19143761 10.1111/j.1365-2184.2008.00572.x PMC6496581

[50] Joe AWB, Yi L, Natarajan A, Le Grand F, So L, et al (2010) Muscle injury activated resident fibro/adipogenic progenitors that facilitate myogenesis. Nat Cell Biol 12: 153–163. 20081841 10.1038/ncb2015 PMC4580288

[51] Ghaffari A, Li Y, Karami A, Ghaffari M, Tredget EE, et al (2006) Fibroblast extracellular matrix gene expression in response to keratinocyte-releasable stratifin. J Cell Biochem 98: 383–393. 16440305 10.1002/jcb.20782

[52] Medina A, Kilani RT, Carr N, Brown E, Ghahary A (2007) Transdifferentiation of periferial blood mononuclear cells into epithelial-like cells. Amer J Pathol 171: 1140–1152. 17717137 10.2353/ajpath.2007.070051 PMC1988865

[53] Chavez-Munoz C, Kilani RT, Ghahary A (2009) Profile of exosomes related proteins released by differentiated and undifferentiated human keratinocytes. J Cell Physiol 221: 221–231. 19530224 10.1002/jcp.21847

[54] Ecker R, Rogojanu R, Streit M, Oesterreicher K, Steiner G (2006) An improved method for discrimination of cell population in tissue sections using microscopy-based multicolor tissue cytometry. Cytometry Part A 69A: 119–123. 10.1002/cyto.a.20219 16479616

[55] Bailey A, Kapur S, Katz AJ (2010) Characterization of adipose-derive stem cells: an update. Curr Stem Cell Res Ther 5: 95–102. 19941461 10.2174/157488810791268555

[56] Folgiero V, Migliano E, Tedesco M, Iacovelli S, Bon G, et al (2010) Purification and characterization of adipose-derived stem cells from patients with lipoaspirates. Cell Transplant 19: 1225–1235. 21208530 10.3727/09638910X519265

[57] Gimble JM, Katz AJ, Bunnell BA (2007) Adipose-derived stem cells for regenerative medicine. Circ Res 100: 1249–1260. 17495232 10.1161/01.RES.0000265074.83288.09 PMC5679280

[58] Augello A, Kurth T, De Bari C (2010) Mesenchymal stem cells: a perspective from in vitro cultures to in vivo migration and niches. Eur Cell Mater 1: 121–133. 10.22203/ecm.v020a11 21249629

[59] Akbulut H, Cuce G, Aktan T, Duman S (2012) Expression of mesenchymal stem cell markers of human adipose tissue surrounding the vas deferens. Biomedical Research 23: 166–169.

[60] Braun J, Kurtz A, Barutcu N, Bodo J, Thiel A, et al (2013) Concerted regulation of CD34 and CD105 accompanies mesenchymal stromal cell derivation from human adventitial stromal cell. Stem Cells Dev 22: 815–827. 23072708 10.1089/scd.2012.0263

[61] Guilak F, Estes B, Diekman B, Moutos F, Gimble JM (2010) Nicolas Andry Award: Multipotent adult stem cells from adipose tissue for musculoskeletal tissue engineering. Clin Orthop Relat Res 468: 2530–2540. 20625952 10.1007/s11999-010-1410-9 PMC2919887

[62] Wagers A, Weissman I (2004) Plasticity of adult stem cells. Cell 116: 639–648. 15006347 10.1016/s0092-8674(04)00208-9

[63] Young H, Black A (2004) Adult stem cells. Anat Rec Part A 267A: 75–102. 10.1002/ar.a.10134 14699636

[64] Shi J, Fu W, Wang X, Xu Y, Li G, et al.. (2012) Transdifferentiation of human adipose-derived stem cells into urothelial cells: potential for urinary tract tissue engineering. Cell Tissue Res e-published ahead. 10.1007/s00441-011-1317-0 22290635

[65] Stocum D (2002) A Tail of Transdifferentiation. Science 298: 1901–1902. 12471238 10.1126/science.1079853

[66] Watt F (1983) Involucrin and other markers of keratinocyte terminal differentiation. J Invest Dermatol 81: 100s–103s. 6345687 10.1111/1523-1747.ep12540786

[67] Murphy G, Flynn T, Rice R, Pinkus G (1984) Involucrin expression in normal and neoplastic human skin: a marker for keratinocyte differentiation. J Invest Dermatol 82: 453–457. 6392430 10.1111/1523-1747.ep12260945

[68] Paunescu V, Deak E, Herman D, Siska I, Tanasie G, et al (2007) In vitro differentiation of human mesenchymal stem cells to epithelial lineage. J Cell Mol Med 11: 502–508. 17635641 10.1111/j.1582-4934.2007.00041.x PMC3922356

[69] Kerpedjieva S, Kim D, Barbeau D, Tamama K (2012) EGRF ligands drive multipotential stromal cells to produce multiple growth factors and cytokines via early growth response-1. Stem Cells Dev: Epub ahead of print. 10.1089/scd.2011.0711 PMC3424970 22316125

[70] Su M, Bikle D, Mancianti M, Pillai S (1994) 1,25-Dihydroxy vitamin D3 potentiates the keratinocyte response to calcium. J Biol Chem 269: 14723–14729. 7910167

